# Safety and efficacy of anti-inflammatory therapy in patients with coronary artery disease: a systematic review and meta-analysis

**DOI:** 10.1186/s12872-022-02525-9

**Published:** 2022-03-04

**Authors:** Ying Niu, Nan Bai, Ying Ma, Peng-Yu Zhong, Yao-Sheng Shang, Zhi-Lu Wang

**Affiliations:** 1 The First Clinical Medical College of Lanzhou University, Lanzhou, China; 2 Department of Cardiology, The First Hospital of Lanzhou University, No. 1, Donggang West Road, Chengguan District, Lanzhou, 730000 Gansu China

**Keywords:** Coronary artery disease, Anti-inflammatory therapy, Meta-analysis

## Abstract

**Background:**

The inflammation hypothesis of atherosclerosis has been put forward for more than 20 years. Although many animal experiments have suggested that anti-inflammatory therapy can inhibit the atherosclerotic process, the efficacy of anti-inflammatory therapy for patients with coronary artery disease (CAD) is still controversial. Therefore, this study aims to evaluate the safety and efficacy of anti-inflammatory drugs in patients with CAD.

**Method:**

We conducted this systematic review and meta-analysis of randomized controlled trials by searching PubMed, EMBASE, web of science, and Cochrane Library database. The primary outcome was a composite outcome of cardiovascular death, myocardial infarction (MI), or stroke. The secondary outcomes included individual MI, coronary revascularization, cardiovascular death, all-cause death, and stroke. The relative risk (RR) and 95% confidence intervals (CI) for outcome events were calculated by the fixed effects model, and trial sequential analysis was applied to assess the results.

**Result:**

A total of ten randomized controlled trials and 60,782 patients with CAD was included. Compared with patients receiving placebo, anti-inflammatory therapy significantly reduced the incidence of the primary outcome in patients with CAD (RR 0.93, 0.89–0.98, *P* = 0.007). In addition, the anti-inflammatory therapy can also reduce the risk of MI (RR 0.90, 0.84–0.96, *P* = 0.002) and coronary revascularization (RR 0.74, 0.66–0.84, *P* < 0.00001) remarkably. However, there was no significant difference in the incidence of cardiovascular death (RR 0.94, 0.86–1.02, *P* = 0.14), all-cause death (RR 1.00, 0.94–1.07, *P* = 0.98) and stroke (RR 0.96, 0.85–1.09, *P* = 0.51) between two groups.

**Conclusions:**

Anti-inflammatory therapy can reduce the incidence of the primary outcome in patients with CAD, especially the risk of MI and coronary revascularization. However, anti-inflammatory therapy increases the risk of infection. (Registered by PROSPERO, CRD 420212291032).

**Supplementary Information:**

The online version contains supplementary material available at 10.1186/s12872-022-02525-9.

## Background

Chronic low-grade inflammation plays an important role in the development of atherosclerosis. However, atherosclerosis is the pathological basis of coronary artery disease (CAD), which can further increase the risk of cardiovascular events, including death, myocardial infarction (MI), stroke, and even cardiac arrest. Despite the use of traditional medicines and revascularization have significantly improved the net clinical benifits, we still need to find more new treatment strategies to reduce the persistent cardiovascular risk [1].

Based on the central role of the inflammatory process in patients with CAD, targeted anti-inflammatory therapy seems to be a promising strategy to reduce residual cardiovascular risk [2]. The anti-inflammatory effects of statins have been noticed in the early twenty-first century [3], and it could bring clinical benefits for patients with evidence of vascular inflammation [4, 5]. In addition, the positive effect of colchicine on patients with cardiovascular events was first reported in 2007 [6]. Subsequently, many randomized trials explored the role of colchicine as an anti-inflammatory drug in patients with CAD [7–10], which suggests that low-dose colchicine anti-inflammatory therapy has certain benefits for patients with CAD. In addition, the CANTOS trial proved that canakinumab can reduce major cardiovascular adverse events by 15%, which provides the proof of principle for targeting pro-inflammatory cytokine pathways [11]. Meanwhile, varespladib and darapladib are effective drugs to reduce the levels of secretory phospholipase A_2_ (sPLA_2_) and Lipoprotein phospholipase A_2_ (Lp-PLA_2_), respectively. They are associated with active oxidized low-density lipoprotein particles, leading to atherosclerosis and plaque rupture [12, 13]. However, three large-scale trials of lipoprotein-coupled phospholipase A_2_ inhibitors did not prove the cardiovascular benefits of anti-inflammatory therapy [14–16], but the VISTA-16 trial shows that varespladib therapy increased the risk of myocardial infarction [14]. Finally, anti-inflammatory therapy is not recommended by the guidelines in patients with CAD.

Therefore, whether the anti-inflammatory therapy can further reduce cardiovascular risk based on standard drug therapy is still controversial. This systematic review and meta-analysis aimed to analyze the safety and efficacy of anti-inflammatory therapy in patients with CAD. The results showed that the anti-inflammatory therapy is effective for patients with CAD, especially the anti-inflammatory drugs that target the central interleukin-6 (IL-6) inflammatory signaling pathway.

## Method

### Data sources and quality assessment

This systematic review and meta-analysis of randomized controlled trials were reported according to the Preferred Reporting Items for Systematic Review and Meta-Analysis (PRISMA) guideline [17]. PubMed, web of science, EMBASE, and Cochrane Library database as well as other sources were searched from inception to 1, January 2022. The searches strategy of PubMed as follows: “Anti-Inflammation” and “Coronary artery disease” combined text and MeSH terms. We also manually searched references for relevant meta-analyses. There were no language restrictions for retrieval. An update reminder for PubMed was created to keep up with the latest research. The detial search strategies of all database were shown (Additional file 2: Table S1). The inclusion criteria of this study: (a) adults aged ≥ 18 years; (b) randomized controlled trial comparing anti-inflammatory drugs to placebo in patients with CAD; (c) follow-up for at least 6 months; (d) sample size > 200 patients; (e) availability of complete clinical outcome data. The exclusion criteria included: (a) nonsteroidal anti-inflammatory drugs or drugs that inhibit complement C5; (b) patients with coronary artery bypass grafting received anti-inflammatory therapy; (c) anti-inflammatory drugs for patients with myocarditis, pericarditis, autoimmune disease, and other non-coronary artery diseases. In this meta-analysis, two investigators (Ying Niu and Nan Bai) independently screened all titles and abstracts, full-text articles of relevant trials, and then evaluated the eligibility of the trials following the inclusion and exclusion criteria. The disagreement was discussed to resolve by a third party (Ying Ma, Peng-yu Zhong, and Yao-sheng Shang). The risk of bias for each trial was assessed by the Cochrane tool of collaboration, and the quality of evidence for each outcome was evaluated by the Grades of Recommendations Assessment Development and Evaluation (GRADE) [18, 19]. The clinical protocols of all included trials were approved by local ethics and informed consent of patients was obtained. The meta-analysis protocol was registered in PROSPERO (CRD 420212291032).

### Data acquisition and clinical outcomes

Two investigators jointly extracted the characteristics of each trial including the baseline characteristics of patients, and the outcome of each trial. Differences should be settled by a third party through consultation (Zhi-lu Wang). The primary outcome was a composite outcome of cardiovascular death, MI, or stroke. The secondary outcomes included MI, coronary revascularization, cardiovascular death, all-cause death, and stroke. Meanwhile, we performed any serious adverse event, infection, and any cancer as a safety outcome. Coronary revascularization is defined as urgent or ischemia-driven coronary revascularization, MI included nonfatal myocardial infarction, ST-segment elevation myocardial infarction, or non-ST-segment elevation myocardial infarction. In addition, based on the definition of clinical studies’ cardiovascular death, all-cause death, stroke, and the safety outcomes of any serious adverse event, infection, and any cancer were defined.

### Statistical analysis

ReviewManager 5.4 (The Nordic Cochrane Center, Copenhagen, Denmark) and Stata version 14.0 were used for all data analysis. The statistical significance was set to *P* < 0.05. The risk ratio (RR) and 95% confidence interval (CI) of each outcome were calculated by fixed-effects model and Mantel–Haenszel method, and Pearson chi-square test and Higgins *I*^*2*^ test were employed to assess the heterogeneity of Cochrane Q statistics. When there was significant heterogeneity (*P*-value of chi-square test was < 0.10) among studies, *I*^*2*^ was used to judge the degree of heterogeneity, and the sources of heterogeneity were found through sensitivity analysis and subgroup analysis. Meanwhile, the sensitivity analysis was used to test the impacts of any individual study for overall results. The Cochrane Collaborative Institutional Risk Bias Assessment Tool was applied to appraise the quality of each randomized controlled trial [18]. In addition, the Egger’s and Bgge’s tests were used to assess the publication bias. Meanwhile, Trial Sequential Analysis version 0.9.5.10 software (Copenhagen Trial Unit, CTU) was conducted to assess the results and conculate the sample size.

## Results

### Search results and study characteristics

**Fig. 1:**
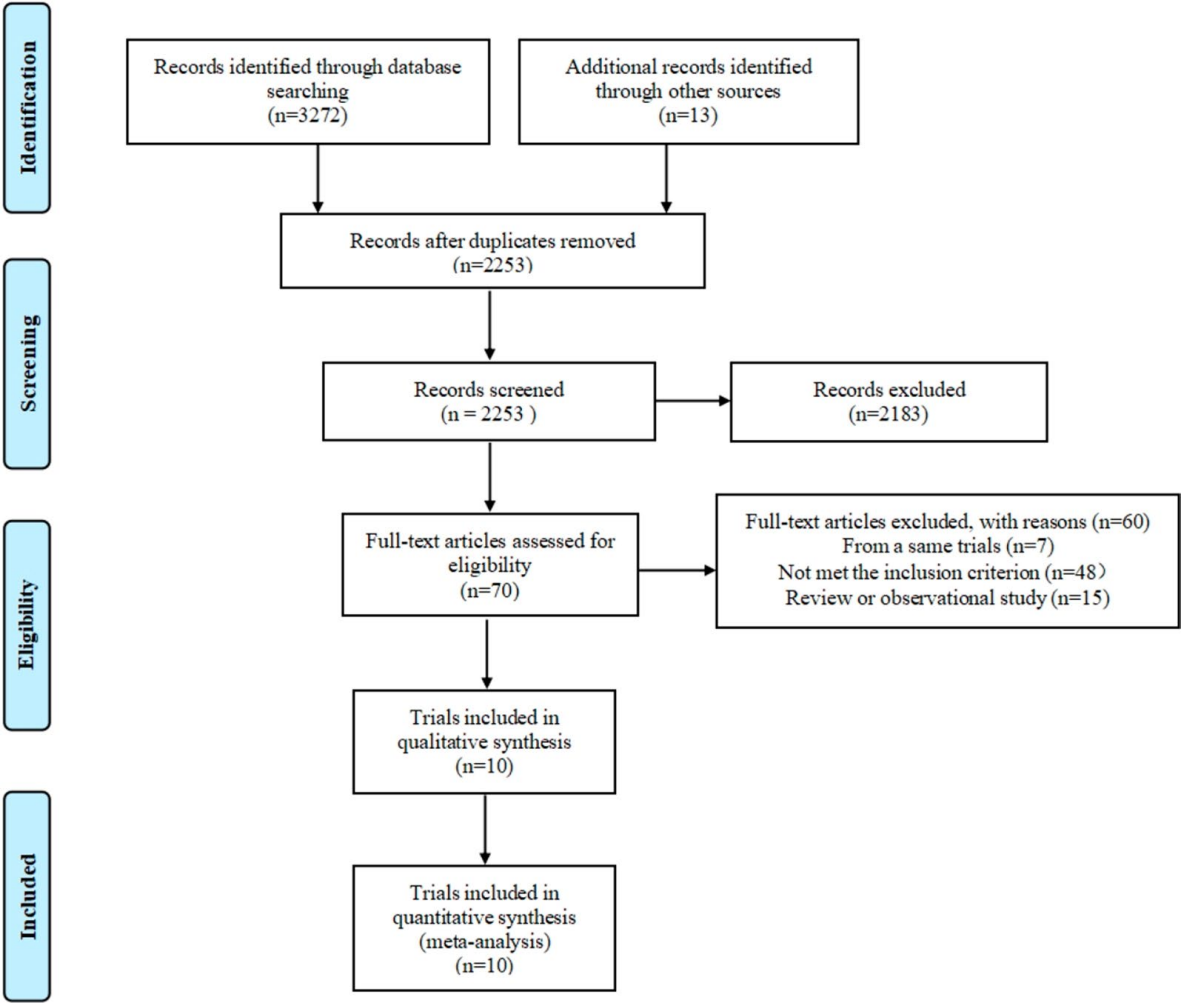
Flow diagram of literature search

A total of 2077 articles were retrieved from medical databases, and 8 articles were from references of relevant reviews. Among them, 1335 articles were identified by reading the title and abstract, and 70 articles were identified by reading the full text. Finally, ten randomized controlled trials involving 60,782 patients with CAD (32,065 patients received the anti-inflammatory therapy and 26,674 patients received placebo) are included (Fig. 1).

The characteristics of the included trials were shown (Table 1). Four trials involved colchicine [7–9, 20]. In addition, four trials compared PLA_2_ inhibitors [14–16, 21], of which three compared varespladib, one compared darapladib. The remaining two trials compared low-dose canakinumab and methotrexate with placebo, respectively [11, 22]. Meanwhile, eight of them included patients with acute coronary syndrome, and three recruited patients with chronic coronary syndrome. The duration of follow-up in the trials ranged from 6 to 48 months.

**Table 1:** Baseline characteristics of the included trials

Study	Publication year	Type	Study cohort	Study totol size	Randomization			Follow up (month)
Mehdi Akrami et al. [7]	2021	RCT	ACS	249	Colchicine (n = 120)	VS	Placebo (n = 129)	6
LoDoCo2 [9]	2020	RCT	CCS	5522	Colchicine (n = 2762)	VS	Placebo (n = 2760)	28.6
CIRT [22]	2018	RCT	MI and MS OR T2MD	4786	Methotrexa (n = 2391)	VS	Placebo (n = 2395)	27.6
COLCOT [8]	2019	RCT	MI	4745	Colchicine (n = 2366)	VS	placebo (n = 237)	22.6
CANTOS [11]	2017	RCT	MI	10,061	Canakinumab (n = 6717)	VS	placebo (n = 3344)	48
STABILITY [15]	2014	RCT	CCS	15,828	Darapladib (n = 7924)	VS	placebo (n = 7904)	44.4
SOLID-TIMI [16]	2014	RCT	ACS	13,026	Darapladib (n = 6504)	VS	placebo (n = 6522)	30
VISTA-16 [14]	2013	RCT	ACS	5145	Varespladib (n = 2572)	VS	placebo (n = 2673)	6
COPS [20]	2020	RCT	ACS	795	Colchicine (n = 396)	VS	placebo (n = 399)	12
FRANCIS [21]	2010	RCT	ACS	625	Varespladib (n = 313)	VS	placebo (n = 311)	6

RCT, randomized controlled trial; CCS, chronic coronary syndrome; MI, myocardial infarction; MS, metabolic syndrome; T2MD, Type 2 diabetes mellitus; ACS, acute coronary syndrome

The baseline characteristics of patients were shown (Table 2). The average age of patients in the anti-inflammatory therapy group was approximately 61.8 years old and about 78.8% patients were male. In addition, 28.3% patients had diabetes, 67.6% patients accompanied hypertension, 41.8% patients suffered from PCI, and 25.5% patients had a history of current smoking. Meanwhile, the average age of patients was approximately 62.0 years old in the placebo group, of which 78.8% patients were male. Furthermore, 25.9% patients had diabetes, 68.8% patients had hypertension, 42.3% patients received PCI, and 27.8% patients had a history of current smoking approximately. Finally, in terms of optimal medical therapy, 94.6% and 95.7% the patients in the anti-inflammatory therapy group received antiplatelet and statin, respectively. Meanwhile, the antiplatelet and statin therapy rates in the placebo group were 93.5% and 95.9%. In addition, in the anti-inflammatory therapy group, 82.9% of the patients used ACEI or ARB, and 83.1% patients used beta-blockers, while 81.9% ACEI or ARB, and 83.8% beta-blockers was used in patients with receiving placebo. The duration of followed-up was 6 to 48 months.

**Table 2:** Baseline characteristics of the patients included

	Mehdi Akrami et al. [7]	LoDoCO2 [9]	CIRT [22]	COLCOT [8]	CANTOS [11]	STABILITY [15]	SOLID-TIMI [16]	VISTA-16 [14]	COPS [20]	FRANCIS [21]
Patients (n)	120/129	2762/2760	2391/2395	2366/2379	3344/6717	7924/7904	6504/6522	2572/2573	396/399	313/311
Age (mean)	56.9/56.9	65.8/65.9	65.6 /66.0	60.6/60.5	61.1/61.1	65.0/65.0	64.0/64.0	61.0/60.7	59.7/60.0	58.5/59.6
Male (%)	86.0/87.0	83.5/85.9	80.7/81.8	80.1/81.6	74.1/74.4	81.5/81.0	74.6/74.5	73.1/74.4	81.3/77.7	73.5/75.9
Smokers (%)	52.0/49.0	11.5/12.0	11.2/11.3	29.9/29.8	22.9/43.1	19.8/21.0	18.9/19.1	33.2/33.4	32.3/37.3	23.3/21.9
Hypertension (%)	52.0/59.0	51.4/50.3	90.0 /90.6	50.1/52.0	79.1/79.9	–	73.7/73.0	74.3/76.8	50.8/49.9	86.6/88.1
Diabetes (%)	27.0/32.0	17.8/18.7	33.0/34.4	19.5/20.9	39.9/40.1	33.6/34.0	35.0/34.1	31.1/31.2	18.9/19.8	26.8/28.0
Previous ACS (%)	–	84.1/84.6	60.7/60.9	15.6/16.7	87.9/88.2	59.1/69.1	31.0/31.2	29.6/30.2	59.0/59.0	–
Previous PCI (%)	16.0/20.0	76.0/75.3	58.4/59.3	16.6/17.0	65.6/67.3	50.3/50.3	23.6/24.2	18.6/17.7	51.0/50.0	–
Previous CABG (%)	4.0/3.0	11.5/14.2	42.2/43.1	2.9/3.4	14.0/14.0	33.4/32.8	–	7.1/6.3	15.0/19.0	–
*Medication use — no. (%)*										
Antiplatelet	100.0/100.0	90.0/80.6	87.1/85.8	98.6/98.9	91.1/92.1	100.0/100.0	96.4/96.5	91.3/91.8	99.0/98.0	92.7/91.0
ACEI or ARB	100.0/100.0	72.2/71.2	72.6/72.0	/	79.6/79.8	–	82.7/82.4	82.5/82.3	88.0/86.0	85.9/81.7
Beta-blocker	100.0/100.0	61.3/62.9	78.2/79.5	89.4/88.3	–	–	87.2/87.4	83.9/82.9	81.0/85.0	84.0/84.6
Statins	100.0/100.0	93.9/94.0	86.1/85.7	98.9/99.1	91.1/91.1	100.0/100.0	94.3/94.9	98.7/99.0	98.0/99.0	–
***Median lipid levels (IQR) — mg/dl***										
LDL cholesterol	–	–	68.0/68.0	–	82.8/82.0	–	74.9/74.9	105.1/105.0	–	61.0/60.3
HDL cholesterol	–	–	41.0/41.0	–	44.5/43.7	44.4/44.8	–	43.2/43.3	–	–

ACS, acute coronary syndrome; PCI, percutaneous coronary intervention; CABG, coronary artery bypass grafting; ACEI, angiotensin-converting enzyme inhibitors; ARB, angiotensin receptor blockers; IQR, inter quartile range

### The primary outcome

**Fig. 2:**
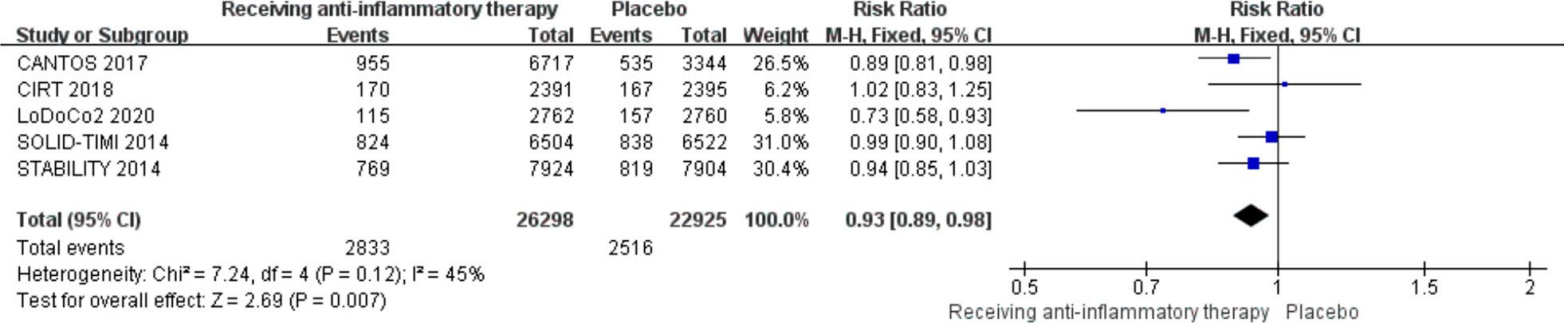
Comparison of the primary outcome between anti-inflammatory therapy and placebo groups

Five trials reported data of the primary outcome, the result showed that the incidence of primary outcome in patients receiving anti-inflammatory therapy was lower than that in patients receiving placebo (10.8% vs 11.0%, RR 0.93, 0. 89–0.98, *P* = 0.007, *I*^*2*^ = 45%, *P*_*h*eterogeneity_ = 0.12) (Fig. 2).

### The secondary outcomes

**Fig. 3:**
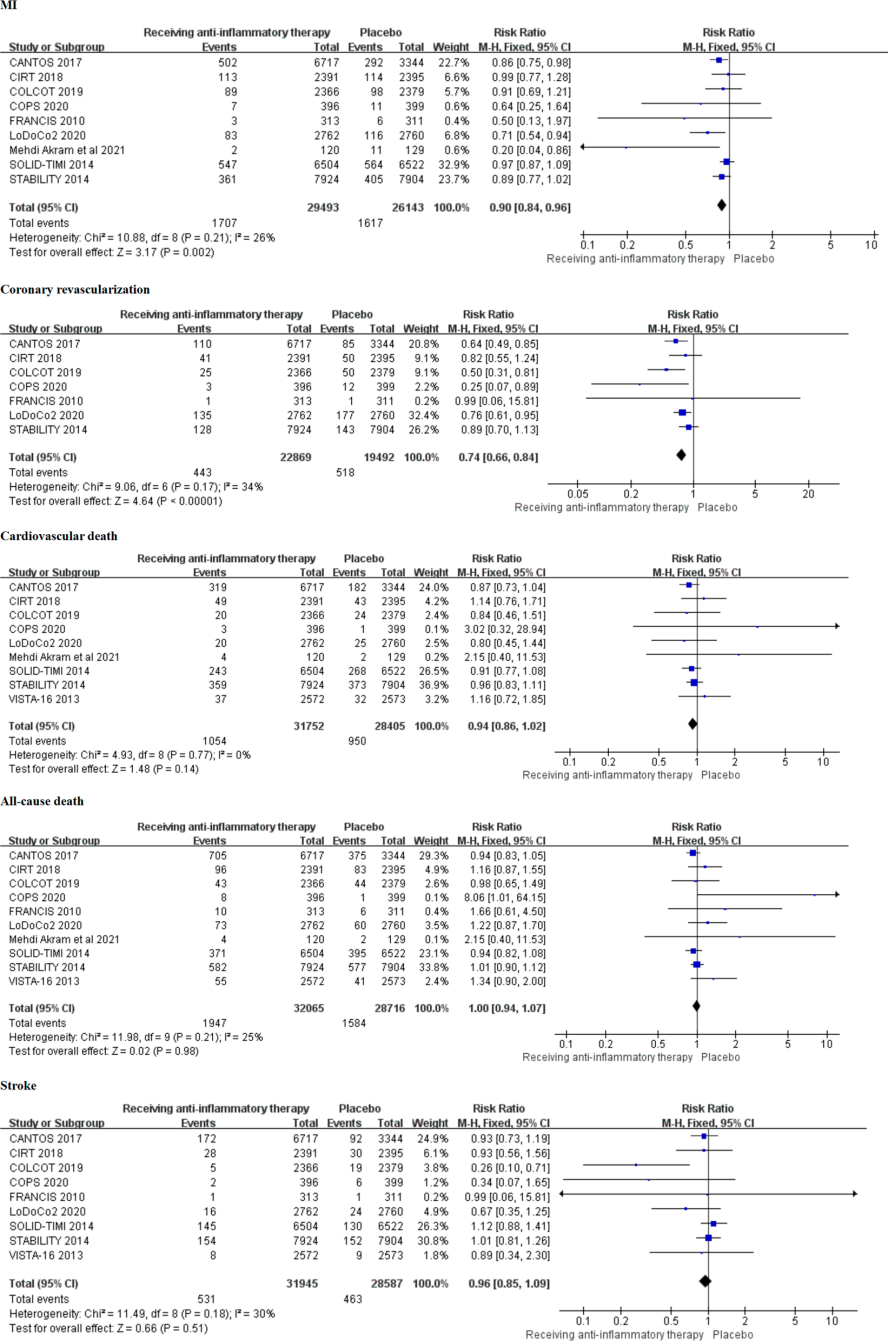
Comparison of the secondary outcomes between anti-inflammatory therapy and placebo groups

The forest map of secondary outcomes was performed (Fig. 3). Nine randomized controlled trials provided the risk of MI in patients with CAD. Compared with patients received placebo, the anti-inflammatory therapy can significantly reduced the risk of MI (5.79% vs 6.19%, RR 0.90, 0.84–0.96, *P* = 0.002, *I*^*2*^ = 26%, *P*_*heterogeneity*_ = 0.21). Meanwhile, the meta-analysis of seven trials displayed that the incidence of coronary revascularization in patients receiving anti-inflammatory therapy was significantly lower than that in patients receiving placebo (1.94% vs 2.66%, RR 0.74, 0.66–0.84,* P* < 0.00001, *I*^*2*^ = 34%, *P*_*heterogeneity*_ = 0.17). Furthermore, the risk of cardiovascular death was reported in eight trials. The result demonstrated that the risk of cardiovascular death was similar between the two groups (3.32% vs 3.34%, RR 0.94, 0.86–1.02, *P* = 0.14, *I*^*2*^ = 0%, *P*_*heterogeneity*_ = 0.77). In addition, there is no significant difference both in the risk of all-cause death (RR 1.00, 0.94–1.07, *P* = 0.98, *I*^*2*^ = 25%, *P*_*heterogeneity*_ = 0.21) and stroke (RR 0.96, 0.85–1.09, *P* = 0.51, *I*^*2*^ = 30%, *P*_*heterogeneity*_ = 0.18) between the two groups.

### The safety outcomes

**Fig. 4:**
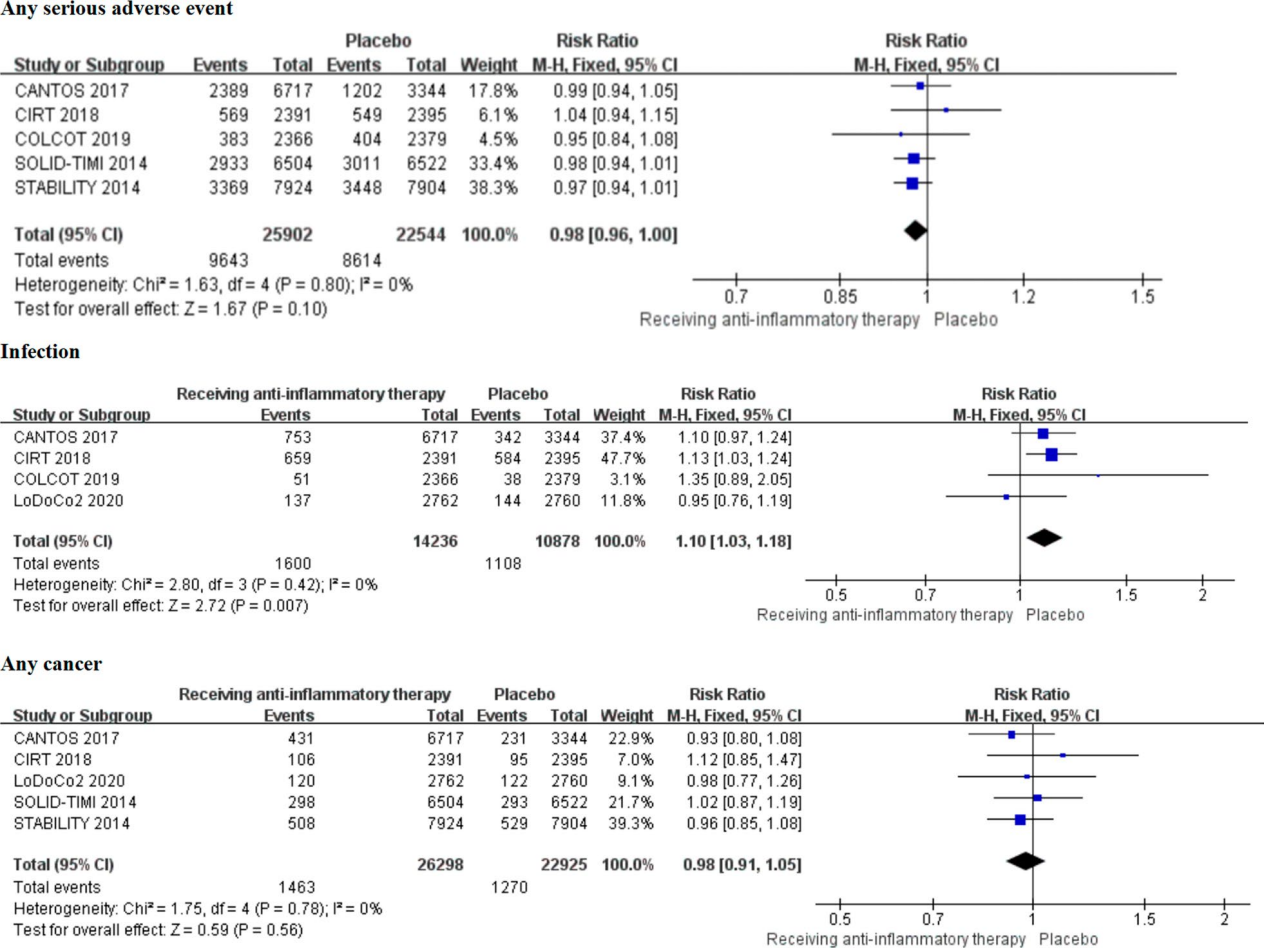
The safety outcomes between anti-inflammatory therapy and placebo groups

Compared placebo group, anti-inflammatory therapy increased the risk of infection in patients of CAD (RR 1.10, 1.03–1.18, *P* = 0.007, *I*^*2*^ = 0%, *P*_*heterogeneity*_ = 0.42). However, no significant difference in incidence of any serious adverse event (RR 0.98, 0.96–1.00, *P* = 0.10, *I*^*2*^ = 0%, *P*_*heterogeneity*_ = 0.80) and any cancer (RR 0.98, 0.91–1.05, *P* = 0.56, *I*^*2*^ = 0%, *P*_*heterogeneity*_ = 0.78) were observed in anti-inflammatory therapy group (Fig. 4).

### Subgroup analysis

**Fig. 5:**
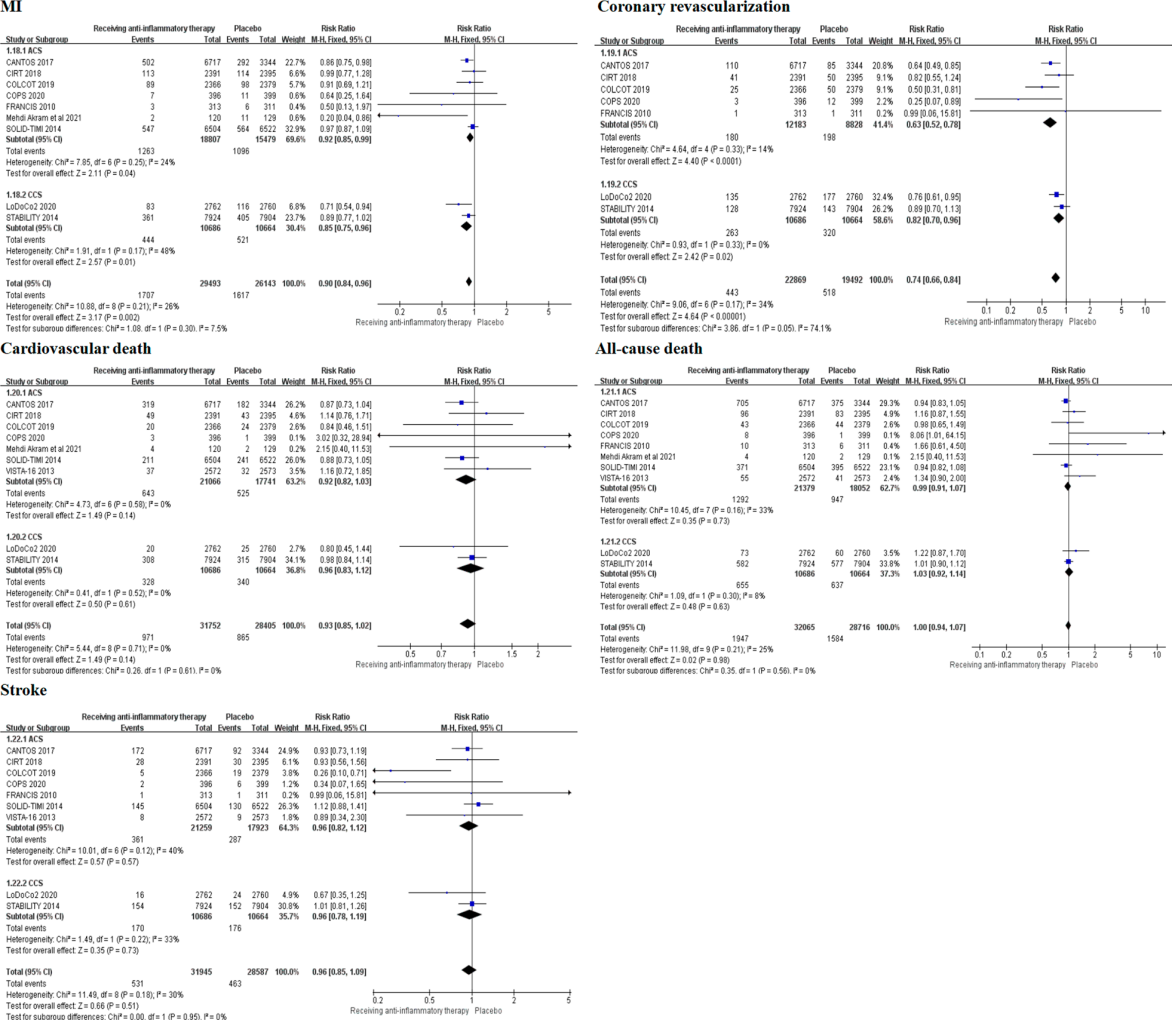
Subgroup analysis of anti-inflammatory therapy in patients with ACS and CCS.ACS, acute coronary syndrome; CCS, chronical coronary syndrome

**Fig. 6:**
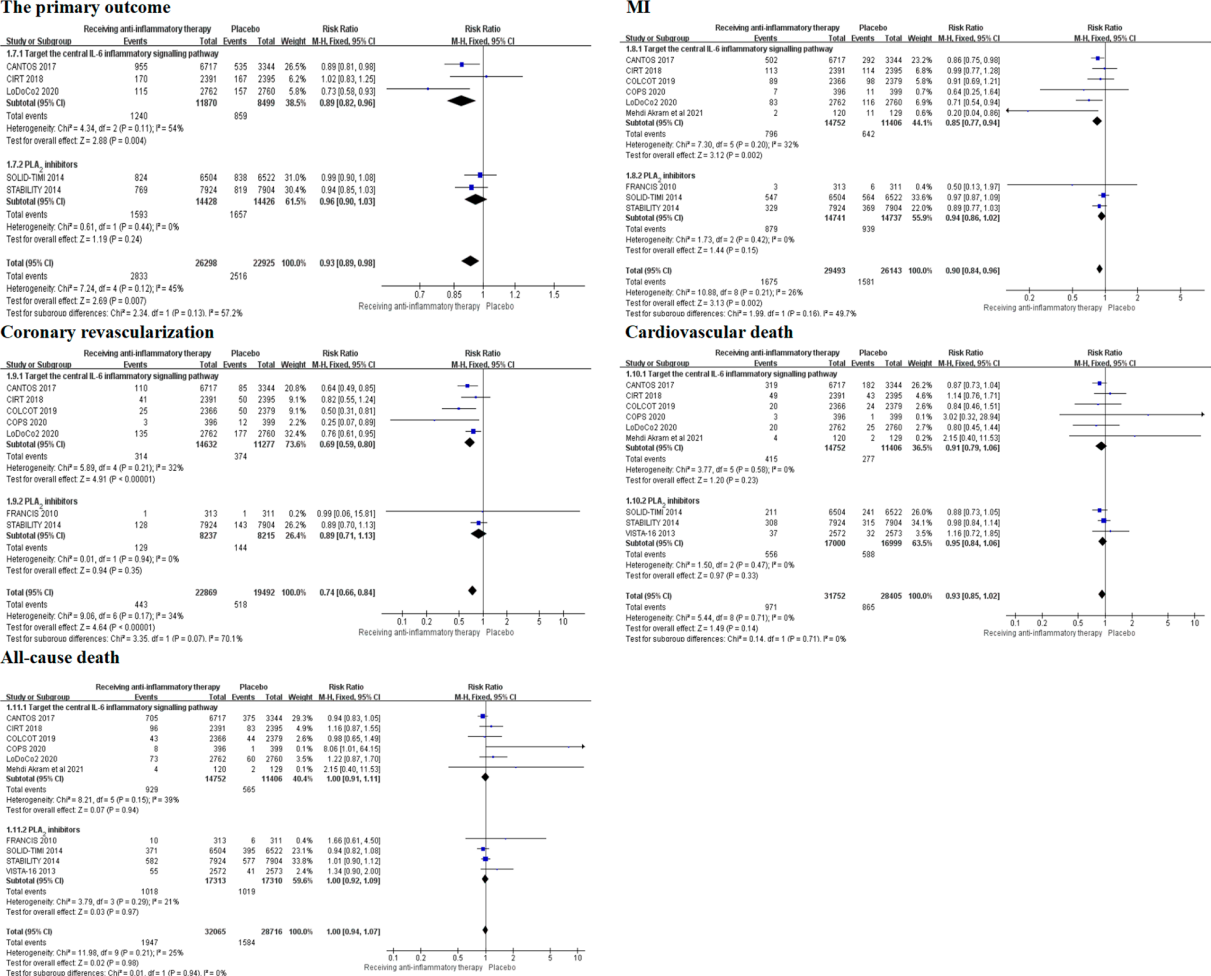
Subgroup analysis of targeting the central IL-6 inflammatory signaling pathway drugs and PLA_2_ inhibitors

We performed subgroup analysis according to the population and type of drugs to explore the most benefit populations and anti-inflammatory drugs in patients with CAD. The subgroup analysis showed that compared with placebo, anti-inflammatory therapy can reduce the risk of coronary revascularization in patients with acute coronary syndrome (RR 0.63, 0.52–0.78, *P* < 0.0001, *I*^*2*^ = 14%, *P*_*heterogeneity*_ = 0.33) and chronic coronary syndrome (RR 0.82, 0.70–0.96, *P* = 0.02, *I*^*2*^ = 0%, *P*_*heterogeneity*_ = 0.33). Meanwhile, there was a significant difference between the two subgroups (*I*^*2*^ = 74.1%, *P*_*interaction*_ = 0.05). However, there was no significant difference between the two subgroups in MI (*I*^*2*^ = 7.5%, *P*_*interaction*_ = 0.3), cardiovascular death (*I*^*2*^ = 0%, *P*_*interaction*_ = 0.61), all-cause death (*I*^*2*^ = 0%, *P*_*interaction*_ = 0.56) and stroke (*I*^*2*^ = 0%, *P*_*interaction*_ = 0.95) (Fig. 5). In addition, another subgroup analysis was performed by the different type of anti-inflammatory drugs. According to the Mendelian randomization data, anti-inflammatory drugs were divided into two categories [23]. Six of ten trials used anti-inflammatory drugs targeting the central IL-6 inflammatory signaling pathway and the other four apply PLA_2_ inhibitors. The results showed that compared with placebo, the anti-inflammatory drugs targeting the central IL-6 inflammatory signaling pathway can reduce the risk of coronary revascularization (RR 0.69 0.59–0.80, *P* < 0.00001, *I*^*2*^ = 32%, *P*_*heterogeneity*_ = 0.21). While, there was no significant difference in PLA_2_ inhibitors subgroup (RR 0.89 0.71–1.13, *P* = 0.35, *I*^*2*^ = 0%, *P*_*heterogeneity*_ = 0.94), and the differences between two groups were statistically significant (*I*^*2*^ = 70.1%, *P*_*interaction*_ = 0.07). However, there is no significant difference in the risk of primary outcome (*I*^*2*^ = 57.2%, *P*_*interaction*_ = 0.13), MI (*I*^*2*^ = 49.7%, *P*_*interaction*_ = 0.16), cardiovascular death (*I*^*2*^ = 0%, *P*_*interaction*_ = 0.71) and all-cause death (*I*^*2*^ = 0%, *P*_*interaction*_ = 0.94) between the two groups (Fig. 6). Further subgroup analysis of colchicine and other drugs targeting the central IL-6 inflammatory signaling pathway showed that colchicine can significantly reduce the incidence of ischemic stroke (RR 0.48 0.29–0.79, *P* = 0.004, *I*^*2*^ = 20%, *P*_*heterogeneity*_ = 0.29) compared with other drugs. There was statistically significant observed in two subgroups (*I*^*2*^ = 82.5%, *P*_*interaction*_ = 0.02) (Additional file 1: Figure S1).

### Trial sequential analysis, assessment of quality and publication bias

Trial sequential analysis was performed for each outcome (Additional file 1: Figure S2). The curve of the primary outcome, MI, and infection exceeded the traditional boundary and the trial sequential analysis boundary. Meanwhile, coronary revascularization exceeded the traditional boundary. However, the curve of cardiovascular death and any serious adverse event did not reach the traditional boundary and the trial sequential analysis boundary. The graph of all-cause death, stroke, and any cancer was generation failed. The risk of bias assessment showed that there was a high risk of bias in attrition (Additional file 1: Figure S3). The quality of GRADE evidence for the primary outcome, coronary revascularization, cardiovascular death, and all-cause death were moderate, while the quality of evidence for MI and stroke outcomes were low (Additional file 2: Table S2). The Egger’s and Begg’s tests were used to assess the publication bias (Additional file 2: Table S3). The *P*-value of other outcomes were more than 0.05 except for MI (Egger’s = 0.04), cardiovascular death (Egger’s = 0.004) and stroke (Egger’s = 0.045). Furthermore, we used the trim and fill method to assessed the impact of publication bias on MI, cardiovascular death, and stroke (Additional file 1: Figure S4).

## Discussion

The findings of this meta-analysis indicate that anti-inflammatory therapy was associated with a lower incidence of primary outcome, MI, and coronary revascularization in patients with CAD. However, there is no significant difference in the risk of cardiovascular death, all-cause death, and stroke. However, anti-inflammatory therapy increased the risk of infection in patients with CAD. Meanwhile, there did not significantly increase the incidence of any serious adverse events and any cancer. In addition, the GRADE evidence levels of outcome for primary outcome, coronary revascularization, infection are moderate, and MI is low according to the certainty of the evidence.

Based on the subgroup analysis, the risk of coronary revascularization was reduced by 31% in the group of targeting the central IL-6 inflammatory signaling pathway and decreased by 37% in patients with acute coronary syndrome group. According to the results of the trial sequential analysis, false-positive results were obtained for coronary revascularization, therefore, more randomized controlled trials are needed to prove these results. In addition, anti-inflammatory therapy can also reduce the incidence of the primary outcome and MI in patients with CAD and the conclusion was reliable. The Egger’s test showed that MI, cardiovascular death, and stroke have publication bias. While the funnel plot has no obvious asymmetry after the trim and fill method.

A recently published meta-analysis of the efficacy of colchicine demonstrated that compared with the placebo group, the colchicine reduced the risk of major adverse cardiovascular events and was not associated with an increased risk for hospitalization, infection risk of common pneumonia, gastrointestinal disorders, and new cancer [24]. Colch icine is a drug targeting the central IL-6 inflammatory signaling pathway. The subgroup analysis of our study showed that the anti-inflammatory drugs targeting the central IL-6 inflammatory signaling pathway can reduce the incidence of primary outcome (composite outcome of cardiovascular death, MI, or stroke), as well as the risk of MI and coronary revascularization. Further subgroup analysis of the drugs targeting the central IL-6 inflammatory signaling pathways showed that colchicine can reduce the incidence of isc hemic stroke to more extent. Another meta-analysis by Haiming Wang, et al. of anti-inflammatory therapy in patients with CAD showed that anti-inflammatory therapy appears to have a beneficial effect on reducing the risk of recurrent myocardial infarction in patients with stable coronary heart disease at the cost of increasing infection [25]. Different from Haiming Wang’s study, we investigated the effect of anti-inflammatory therapy on long-term outcomes in patients with CAD. Our study showed that anti-inflammatory therapy can reduce the incidence of primary outcome, MI, and coronary revascularization in patients with CAD after at least 6 months of follow up, and our study also shows that anti-inflammatory therapy can significantly reduce the incidence of coronary revascularization in patients with acute coronary syndrome.

The results of this meta-analysis need to be applied with caution. Firstly, according to the subgroup analysis of this study, drugs targeting the central IL-6 inflammatory signaling pathway, such as colchicine, canakinumab, and methotrexate, can reduce cardiovascular events in patients with CAD, while PLA2 inhibitors cannot. Therefore, it is recommended that patients with CAD should use anti-inflammatory drugs that inhibit the central IL-6 inflammatory signaling pathway. Meanwhile, colchicine is easy to obtain and economical compared with canakinumab and methotrexate, which improves the compliance of patients. Secondly, patients with chronic coronary syndrome and acute coronary syndrome were included in this study. The results support lower coronary revascularization rates after anti-inflammatory therapy in patients with the acute coronary syndrome. In addition, anti-inflammatory therapy can increase the incidence of infection in patients with CAD. Therefore, it should be used with caution in patients with CAD at high risk of infection. Finally, other factors need to be considered in clinical practice. The characteristics of race are essential factors influencing the effect of anti-inflammatory therapy. The trial by Irena tepanikova et al. showed that the concentrations of inflammation markers in black patients were higher than that in white patients, which led to that black patients may benefit more from anti-inflammatory therapy [26]. However, it should be noted that white people are the majority of participants in this study, and the efficacy of anti-inflammatory therapy in non-white patients needs further studied.

## Limitations

This systematic review and meta-analysis of randomized clinical trials may have some limitations. Firstly, the follow-up duration of all included trials was at least 6 months, the short-term clinical benifts of anti-inflammatory therapy needs further exploration. Secondly, the three small sample size trials had a low incidence of positive events and a wide confidence interval, which reduced the quality of evidence [7, 20, 21]. Thirdly, the lost follow-up rate of three trials was more than 20%, which reduced the reliability of the analysis results [16, 21, 22]. In addition, we cannot obtain the optimal medical therapy, including antiplatelet, statins, beta-blockers, and renin–angiotensin–aldosterone system receptor inhibitor cannot be further analyzed. Finally, the composite outcome of cardiovascular death, MI, and stroke favored the anti-inflammatory group. However, given that the incidence of serious adverse events in the two groups is almost the same, the clinical importance is debatable. Therefore, more randomized trials are needed to prove this.

## Conclusions

Based on standard medical therapy, anti-inflammatory therapy can significantly reduce the incidence of a composite outcome of cardiovascular death, MI, or stroke, MI, and coronary revascularization in patients with CAD, which proves that anti-inflammatory drugs have clinical benefits. However, anti-inflammatory therapy increases the risk of infection, which limited use in patients at high risk of infection. In addition, compared with other anti-inflammatory drugs mentioned in this article, colchicine is more effective in reducing the risk of ischemic stroke. Furthermore, colchicine is cheap and available all over the world, which enables patients to have better compliance.

## Supplementary Information

**Additional file 1. Supplementary Figure 1.** Subgroup analysis of colchicine and other drugs targeting the central IL-6 inflammatory signaling pathway. **Supplementary Figure 2.** Size of information required for each outcome. **Supplementary Figure 3.** Assessment for the risk of bias in each randomized controlled trial included. **Supplementary Figure 4.** The trim and fill method of MI, cardiovascular death, and stroke.

**Additional file 2. Supplementary Table 1.** Search strategy of this meta-analysis. **Supplementary Table 2.** Summary of GRADE evidence quality for each outcome. **Supplementary Table 3.** The P-value of Begg’s and Egger’s for each outcome.

## Data Availability

All data generated or analyzed during this study are included in this published article.

## References

[1] Golia E, Limongelli G, Natale F (2014). Inflammation and cardiovascular disease: from pathogenesis to therapeutic target. Curr Atheroscler Rep.

[2] Libby P, Ridker PM, Maseri A (2002). Inflammation and atherosclerosis. Circulation.

[3] Albert MA, Danielson E, Rifai N (2001). Effect of statin therapy on C-reactive protein levels: the pravastatin inflammation/CRP evaluation (PRINCE): a randomized trial and cohort study. JAMA.

[4] Ridker PM, Danielson E, Fonseca FA (2008). Rosuvastatin to prevent vascular events in men and women with elevated C-reactive protein. N Engl J Med.

[5] Ridker PM, Danielson E, Fonseca FA (2009). Reduction in C-reactive protein and LDL cholesterol and cardiovascular event rates after initiation of rosuvastatin: a prospective study of the JUPITER trial. Lancet.

[6] Crittenden DB, Lehmann RA, Schneck L (2012). Colchicine use is associated with decreased prevalence of myocardial infarction in patients with gout. J Rheumatol.

[7] Akrami M, Izadpanah P, Bazrafshan M (2021). Effects of colchicine on major adverse cardiac events in next 6-month period after acute coronary syndrome occurrence; a randomized placebo-control trial. BMC Cardiovasc Disord.

[8] Tardif JC, Kouz S, Waters DD (2019). Efficacy and safety of low-dose colchicine after myocardial infarction. N Engl J Med.

[9] Nidorf SM, Fiolet ATL, Mosterd A (2020). Colchicine in patients with chronic coronary disease. N Engl J Med.

[10] Vaidya K, Arnott C, Martínez GJ (2018). Colchicine therapy and plaque stabilization in patients with acute coronary syndrome: a CT coronary angiography study. JACC Cardiovasc Imaging.

[11] Ridker PM, Everett BM, Thuren T (2017). Antiinflammatory therapy with canakinumab for atherosclerotic disease. N Engl J Med.

[12] Rosenson RS (2010). Phospholipase A2 inhibition and atherosclerotic vascular disease: prospects for targeting secretory and lipoprotein-associated phospholipase A2 enzymes. Curr Opin Lipidol.

[13] Thompson A, Gao P, Orfei L (2010). Lipoprotein-associated phospholipase A2 and risk of coronary disease, stroke, and mortality: collaborative analysis of 32 prospective studies. Lancet.

[14] Nicholls SJ, Kastelein JJ, Schwartz GG (2014). Varespladib and cardiovascular events in patients with an acute coronary syndrome: the VISTA-16 randomized clinical trial. JAMA.

[15] White HD, Held C, Stewart R (2014). Darapladib for preventing ischemic events in stable coronary heart disease. N Engl J Med.

[16] O'Donoghue ML, Braunwald E, White HD (2014). Effect of darapladib on major coronary events after an acute coronary syndrome: the SOLID-TIMI 52 randomized clinical trial. JAMA.

[17] Shamseer L, Moher D, Clarke M (2015). Preferred reporting items for systematic review and meta-analysis protocols (PRISMA-P) 2015: elaboration and explanation. BMJ.

[18] Higgins JP, Altman DG, Gøtzsche PC (2011). The Cochrane collaboration's tool for assessing risk of bias in randomised trials. BMJ.

[19] Guyatt GH, Oxman AD, Vist GE (2008). GRADE: an emerging consensus on rating quality of evidence and strength of recommendations. BMJ.

[20] Tong DC, Quinn S, Nasis A (2020). Colchicine in patients with acute coronary syndrome: the Australian COPS randomized clinical trial. Circulation.

[21] Rosenson RS, Hislop C, Elliott M (2010). Effects of varespladib methyl on biomarkers and major cardiovascular events in acute coronary syndrome patients. J Am Coll Cardiol.

[22] Ridker PM, Everett BM, Pradhan A (2019). Low-dose methotrexate for the prevention of atherosclerotic events. N Engl J Med.

[23] (2012) The interleukin-6 receptor as a target for prevention of coronary heart disease: a mendelian randomisation analysis. Lancet 379(9822):1214-1224. 10.1016/S0140-6736(12)60110-X. 10.1016/S0140-6736(12)60110-X PMC3316968 22421340

[24] Fiolet ATL, Opstal TSJ, Mosterd A, Eikelboom JW, Jolly SS, Keech AC, Kelly P, Tong DC, Layland J, Nidorf SM, Thompson PL, Budgeon C, Tijssen JGP, Cornel JH (2021). Efficacy and safety of low-dose colchicine in patients with coronary disease: a systematic review and meta-analysis of randomized trials. Eur Heart J.

[25] Wang H, Jiang M, Li X, Zhao Y, Shao J, Liu Z, Lin L, Xu Q, Wang L, Lu X, Zhang H, Chen Y, Zhang R (2021). Anti-inflammatory Therapies for Coronary Heart Disease: A Systematic Review and Meta-Analysis. Front Cardiovasc Med.

[26] Stepanikova I, Bateman LB, Oates GR (2017). Systemic Inflammation in Midlife: Race Socioeconomic Status and Perceived Discrimination. Amer J Prevent Med.

