# Supplementary material for: Safety and efficacy of anti-inflammatory therapy in patients with coronary artery disease: a systematic review and meta-analysis
Source: BMC Cardiovasc Disord. 2022 Mar 4;22:84. doi: 10.1186/s12872-022-02525-9 (PMC8896203; doi:10.1186/s12872-022-02525-9)
Supplement: Supplementary file 1 — Additional file 1. Supplementary Figure 1. Subgroup analysis of colchicine and other drugs targeting the central IL-6 inflammatory signaling pathway. Supplementary Figure 2. Size of information required for each outcome. Supplementary Figure 3. Assessment for the risk of bias in each randomized controlled trial included. Supplementary Figure 4. The trim and fill method of MI, cardiovascular death, and stroke. [file 12872_2022_2525_MOESM1_ESM.docx]

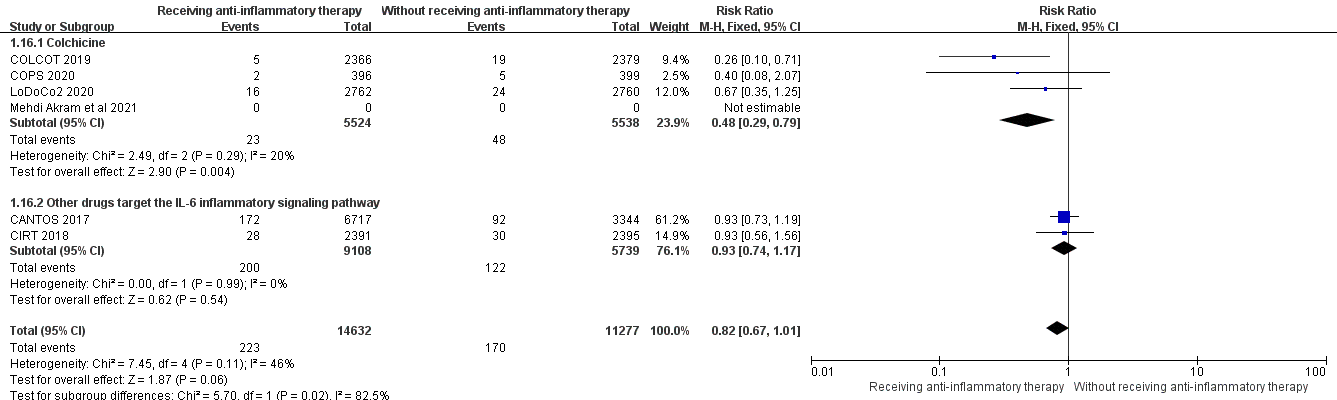


**Supplementary Figure 1.** Subgroup analysis of colchicine and other drugs targeting the central IL-6 inflammatory signaling pathway.


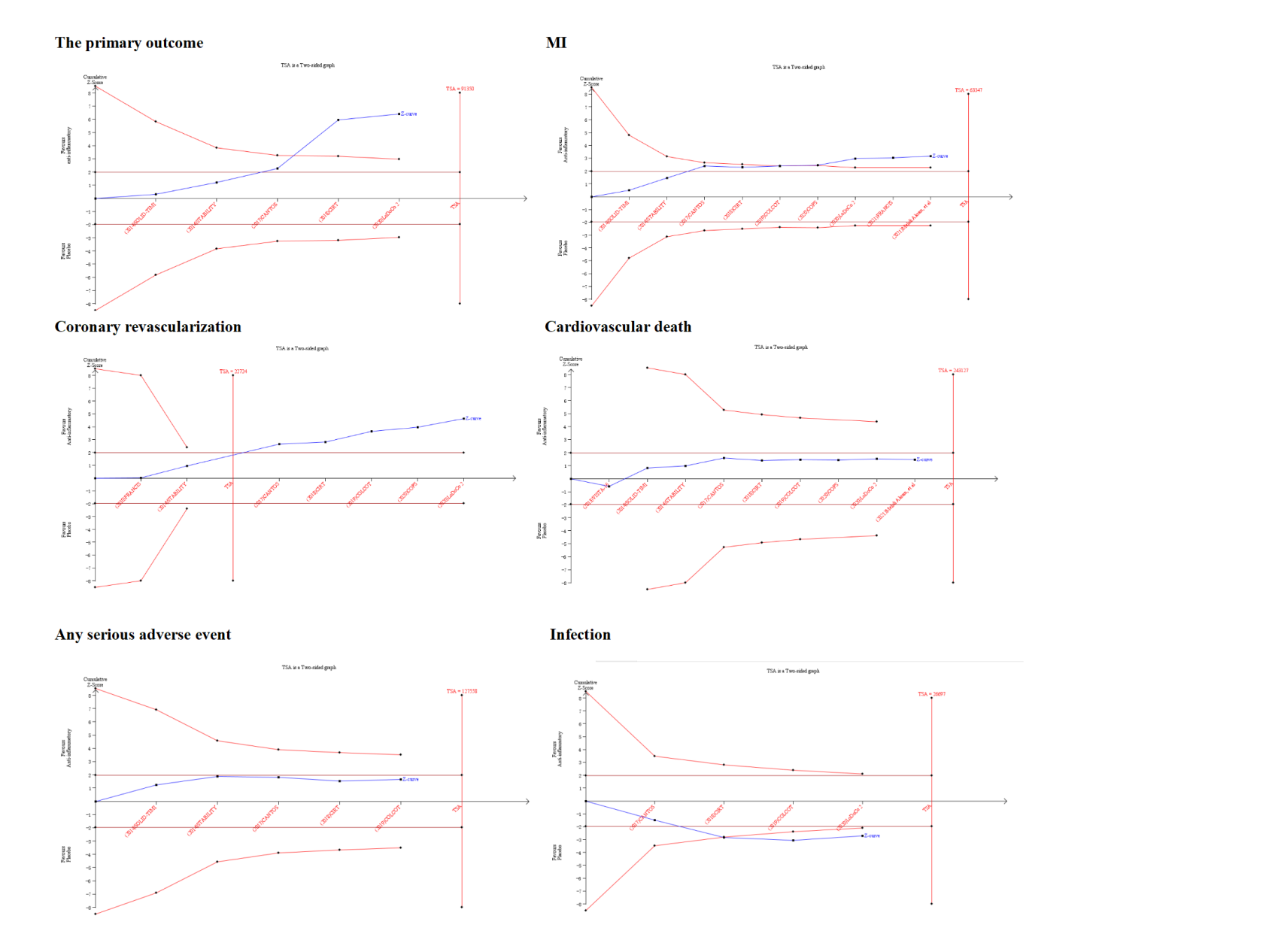


**Supplementary Figure 2.** Size of information required for each outcome.


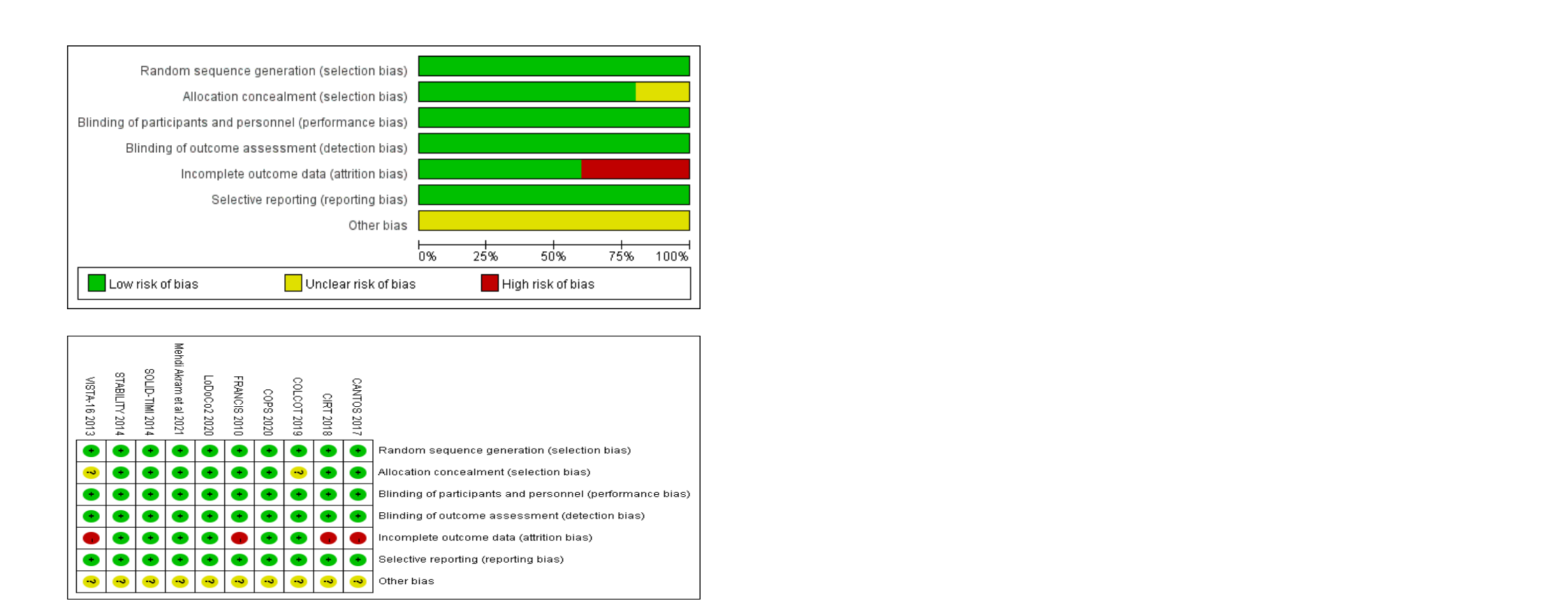


**Supplementary Figure 3.** Assessment for the risk of bias in each randomized controlled trial included.


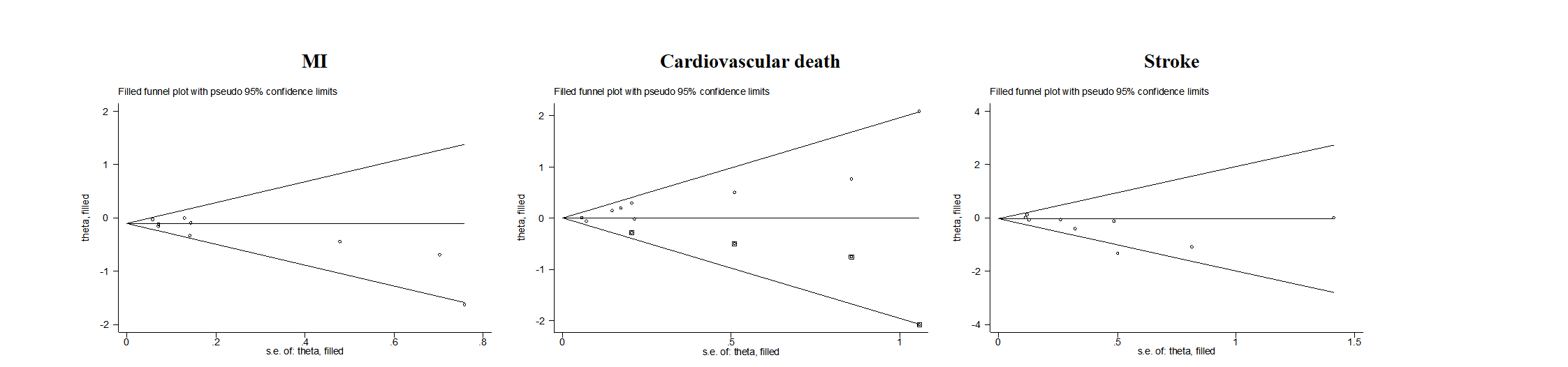


**Supplementary Figure 4**. The trim and fill method of MI, cardiovascular death, and stroke.
