# Supplementary material for: Safety and efficacy of anti-inflammatory therapy in patients with coronary artery disease: a systematic review and meta-analysis
Source: BMC Cardiovasc Disord. 2022 Mar 4;22:84. doi: 10.1186/s12872-022-02525-9 (PMC8896203; doi:10.1186/s12872-022-02525-9)
Supplement: Supplementary file 2 — Additional file 2. Supplementary Table 1. Search strategy of this meta-analysis. Supplementary Table 2. Summary of GRADE evidence quality for each outcome. Supplementary Table 3. The P-value of Begg’s and Egger’s for each outcome. [file 12872_2022_2525_MOESM2_ESM.docx]

**Supplementary Table 1**. Search strategy of this meta-analysis.

| **Searches** | | **Results** |
| --- | --- | --- |
| **Pubmed search strategy** | |  |
| #1 | Search: (((((((((((((anti-inflammatory) OR (Anti Inflammatory Agents)) OR (Antiinflammatory Agent)) OR (Agent, Antiinflammatory)) OR (Antiinflammatory Agents)) OR (Agents, Antiinflammatory)) OR (Anti-Inflammatories)) OR (Anti Inflammatories)) OR (Antiinflammatories)) OR (Anti-Inflammatory Agent)) OR (Agent, Anti-Inflammatory)) OR (Anti Inflammatory Agent)) OR (Agents, Anti-Inflammatory)) OR (Agents, Anti Inflammatory) | 751837 |
| #2 | Search: (((((((((((((((coronary artery disease) OR (Artery Disease, Coronary)) OR (Artery Diseases, Coronary)) OR (Coronary Artery Diseases)) OR (Left Main Coronary Artery Disease)) OR (Left Main Disease)) OR (Left Main Diseases)) OR (Left Main Coronary Disease)) OR (Coronary Arteriosclerosis)) OR (Arterioscleroses, Coronary)) OR (Coronary Arterioscleroses)) OR (Atherosclerosis, Coronary)) OR (Atheroscleroses, Coronary)) OR (Coronary Atheroscleroses)) OR (Coronary Atherosclerosis)) OR (Arteriosclerosis, Coronary) | 223019 |
| #3 | Search: ((((((((((((((anti-inflammatory) OR (Anti Inflammatory Agents)) OR (Antiinflammatory Agent)) OR (Agent, Antiinflammatory)) OR (Antiinflammatory Agents)) OR (Agents, Antiinflammatory)) OR (Anti-Inflammatories)) OR (Anti Inflammatories)) OR (Antiinflammatories)) OR (Anti-Inflammatory Agent)) OR (Agent, Anti-Inflammatory)) OR (Anti Inflammatory Agent)) OR (Agents, Anti-Inflammatory)) OR (Agents, Anti Inflammatory)) AND ((((((((((((((((coronary artery disease) OR (Artery Disease, Coronary)) OR (Artery Diseases, Coronary)) OR (Coronary Artery Diseases)) OR (Left Main Coronary Artery Disease)) OR (Left Main Disease)) OR (Left Main Diseases)) OR (Left Main Coronary Disease)) OR (Coronary Arteriosclerosis)) OR (Arterioscleroses, Coronary)) OR (Coronary Arterioscleroses)) OR (Atherosclerosis, Coronary)) OR (Atheroscleroses, Coronary)) OR (Coronary Atheroscleroses)) OR (Coronary Atherosclerosis)) OR (Arteriosclerosis, Coronary)) | 7017 |
| #4 | Search: ((((((((((((((anti-inflammatory) OR (Anti Inflammatory Agents)) OR (Antiinflammatory Agent)) OR (Agent, Antiinflammatory)) OR (Antiinflammatory Agents)) OR (Agents, Antiinflammatory)) OR (Anti-Inflammatories)) OR (Anti Inflammatories)) OR (Antiinflammatories)) OR (Anti-Inflammatory Agent)) OR (Agent, Anti-Inflammatory)) OR (Anti Inflammatory Agent)) OR (Agents, Anti-Inflammatory)) OR (Agents, Anti Inflammatory)) AND ((((((((((((((((coronary artery disease) OR (Artery Disease, Coronary)) OR (Artery Diseases, Coronary)) OR (Coronary Artery Diseases)) OR (Left Main Coronary Artery Disease)) OR (Left Main Disease)) OR (Left Main Diseases)) OR (Left Main Coronary Disease)) OR (Coronary Arteriosclerosis)) OR (Arterioscleroses, Coronary)) OR (Coronary Arterioscleroses)) OR (Atherosclerosis, Coronary)) OR (Atheroscleroses, Coronary)) OR (Coronary Atheroscleroses)) OR (Coronary Atherosclerosis)) OR (Arteriosclerosis, Coronary)) AND (randomized controlled trial[Filter])) | 792 |
| **EMBASE search strategy.** | |  |
| #1 | coronary AND artery AND disease OR (artery AND disease, AND coronary) OR (artery AND diseases, AND coronary) OR (coronary AND artery AND diseases) OR (left AND main AND coronary AND artery AND disease) OR (left AND main AND diseases) OR (left AND main AND coronary AND disease) OR (coronary AND arteriosclerosis) OR (arterioscleroses, AND coronary) OR (coronary AND arterioscleroses) OR (atherosclerosis, AND coronary) OR (atheroscleroses, AND coronary) OR (coronary AND atheroscleroses) OR (coronary AND atherosclerosis) OR (arteriosclerosis, AND coronary) | 435038 |
| #2 | 'anti inflammatory' OR (anti AND inflammatory AND agents) OR (antiinflammatory AND agent) OR (agent, AND antiinflammatory) OR (antiinflammatory AND agents) OR (agents, AND antiinflammatory) OR 'anti inflammatories' OR (anti AND inflammatories) OR antiinflammatories OR ('anti inflammatory' AND agent) OR (agent, AND 'anti inflammatory') OR (anti AND inflammatory AND agent) OR (agents, AND 'anti inflammatory') OR (agents, AND anti AND inflammatory) | 432529 |
| #3 | (randomized AND controlled AND trial OR randomized OR rct) AND [randomized controlled trial]/lim | 693651 |
| #4 | #1 AND #2 AND #3 | 367 |
| **Cochrane Library search strategy.** | |  |
| #1 | (anti-inflammatory)ti,ab,kw | 23895 |
| #2 | MeSH descriptor [Ant-Inflammatory Agents] this term only | 6107 |
| #3 | MeSH descriptor [Coronary Artery Disease] this term only | 6965 |
| #4 | (coronary artery disease)ti,ab,kw | 25098 |
| #5 | #1 OR #2 | 28395 |
| #6 | #3 OR #4 | 25098 |
| #7 | #5 AND #6 | 447 |
| #8 | RCT OR randomized OR randomized controlled trial | 1249184 |
| #9 | #7 AND 8 | 367 |
| **Web of science search strategy** | |  |
| #1 | TS= (anti-inflammatory OR Anti Inflammatory Agents OR Antiinflammatory Agent OR Agent, Antiinflammatory OR Antiinflammatory Agents OR Agents, Antiinflammatory OR Anti-Inflammatories OR Anti Inflammatories OR Antiinflammatories OR Anti-Inflammatory Agent OR Agent, Anti-Inflammatory OR Anti Inflammatory Agent OR Agents, Anti-Inflammatory OR Agents, Anti Inflammatory) | 251454 |
| #2 | TS= (coronary artery disease OR Artery Disease, Coronary OR Artery Diseases, Coronary OR Coronary Artery Diseases OR Left Main Coronary Artery Disease OR Left Main Disease OR Left Main Diseases OR Left Main Coronary Disease OR Coronary Arteriosclerosis OR Arterioscleroses, Coronary OR Coronary Arterioscleroses OR Atherosclerosis, Coronary OR Atheroscleroses, Coronary OR Coronary Atheroscleroses OR Coronary Atherosclerosis OR Arteriosclerosis, Coronary)) | 249124 |
| #3 | TS= (RCT OR randomized controlled trial OR randomized) | 950996 |
| #4 | #1 AND #2 AND #3 | 551 |

**Supplementary Table 2.** Summary of GRADE evidence quality for each outcome.

| **Safety and efficacy of** **anti-inflammatory therapy in patients with coronary artery disease** | | | | | | |
| --- | --- | --- | --- | --- | --- | --- |
| **Patient or population:** patients with CAD^1^  **Settings:**  **Intervention:** anti-inflammatory therapy | | | | | | |
| **Outcomes** | **Illustrative comparative risks* (95% CI)** | | **Relative effect**  **(95% CI)** | **No of Participants**  **(studies)** | **Quality of the evidence**  **(GRADE)** | **Comments** |
|  | Assumed risk | Corresponding risk |  |  |  |  |
|  | **Control** | **Anti-inflammatory therapy** |  |  |  |  |
| **The primary outcome**  Follow-up: 6-48 months | **Study population** | | **RR 0.93**  (0.89 to 0.98) | 49223  (5 studies) | ⊕⊕⊕⊝  **moderate**^1^ |  |
|  | **110 per 1000** | **102 per 1000**  (98 to 108) |  |  |  |  |
|  | **Moderate** | |  |  |  |  |
|  | **104 per 1000** | **97 per 1000**  (93 to 102) |  |  |  |  |
| **MI**  Follow-up: 6-48 months | **Study population** | | **RR 0.9**  (0.84 to 0.96) | 55636  (9 studies) | ⊕⊕⊝⊝  **low**^1,2^ |  |
|  | **62 per 1000** | **56 per 1000**  (52 to 59) |  |  |  |  |
|  | **Moderate** | |  |  |  |  |
|  | **48 per 1000** | **43 per 1000**  (40 to 46) |  |  |  |  |
| **Coronary revascularization**  Follow-up: 6-48 months | **Study population** | | **RR 0.74**  (0.66 to 0.84) | 42361  (7 studies) | ⊕⊕⊕⊝  **moderate**^1^ |  |
|  | **27 per 1000** | **20 per 1000**  (18 to 22) |  |  |  |  |
|  | **Moderate** | |  |  |  |  |
|  | **21 per 1000** | **16 per 1000**  (14 to 18) |  |  |  |  |
| **Cardiovascular death**  Follow-up: 6-48 months | **Study population** | | **RR 0.94**  (0.86 to 1.02) | 60157  (9 studies) | ⊕⊕⊕⊝  **moderate**^1^ |  |
|  | **33 per 1000** | **31 per 1000**  (29 to 34) |  |  |  |  |
|  | **Moderate** | |  |  |  |  |
|  | **16 per 1000** | **15 per 1000**  (14 to 16) |  |  |  |  |
| **All-cause death**  Follow-up: 6-48 months | **Study population** | | **RR 1**  (0.94 to 1.07) | 60781  (10 studies) | ⊕⊕⊕⊝  **moderate**^1^ |  |
|  | **55 per 1000** | **55 per 1000**  (52 to 59) |  |  |  |  |
|  | **Moderate** | |  |  |  |  |
|  | **21 per 1000** | **21 per 1000**  (20 to 22) |  |  |  |  |
| **Stroke**  Follow-up: 6-48 months | **Study population** | | **RR 0.96**  (0.85 to 1.09) | 60532  (9 studies) | ⊕⊕⊝⊝  **low**^1,2^ |  |
|  | **16 per 1000** | **16 per 1000**  (14 to 18) |  |  |  |  |
|  | **Moderate** | |  |  |  |  |
|  | **13 per 1000** | **12 per 1000**  (11 to 14) |  |  |  |  |
| **Any serious adverse event**  Follow-up: 6-48 months | **Study population** | | **RR 0.98**  (0.96 to 1) | 48446  (5 studies) | ⊕⊕⊕⊝  **moderate**^1^ |  |
|  | **382 per 1000** | **374 per 1000**  (367 to 382) |  |  |  |  |
|  | **Moderate** | |  |  |  |  |
|  | **359 per 1000** | **352 per 1000**  (345 to 359) |  |  |  |  |
| **Infection**  Follow-up: 6-48 months | **Study population** | | **RR 1.06**  (0.99 to 1.14) | 25114  (4 studies) | ⊕⊕⊕⊝  **moderate**^1^ |  |
|  | **99 per 1000** | **105 per 1000**  (98 to 112) |  |  |  |  |
|  | **Moderate** | |  |  |  |  |
|  | **77 per 1000** | **82 per 1000**  (76 to 88) |  |  |  |  |
| **Any cancer**  Follow-up: 6-48 months | **Study population** | | **RR 0.98**  (0.91 to 1.05) | 49223  (5 studies) | ⊕⊕⊕⊝  **moderate**^1^ |  |
|  | **55 per 1000** | **54 per 1000**  (50 to 58) |  |  |  |  |
|  | **Moderate** | |  |  |  |  |
|  | **45 per 1000** | **44 per 1000**  (41 to 47) |  |  |  |  |
| *The basis for the **assumed risk** (e.g. the median control group risk across studies) is provided in footnotes. The **corresponding risk** (and its 95% confidence interval) is based on the assumed risk in the comparison group and the **relative effect** of the intervention (and its 95% CI).  **CI:** Confidence interval; **RR:** Risk ratio; | | | | | | |
| GRADE Working Group grades of evidence  **High quality:** Further research is very unlikely to change our confidence in the estimate of effect.  **Moderate quality:** Further research is likely to have an important impact on our confidence in the estimate of effect and may change the estimate.  **Low quality:** Further research is very likely to have an important impact on our confidence in the estimate of effect and is likely to change the estimate.  **Very low quality:** We are very uncertain about the estimate. | | | | | | |
| ^1^ Lost to follow-up > 20%  ^2^ Egger's test P<0.05 | | | | | | |

**Supplementary Table 3.** The *P* value of Begg’s and Egger’s for each outcome.

|  | *P* value of Begg’s test | *P* value of Egger’s test |
| --- | --- | --- |
| The primary outcome | 0.221 | 0.498 |
| MI | 0.076 | 0.040 |
| Coronary revascularization | 0.230 | 0.261 |
| Cardiovascular death | 0.251 | 0.127 |
| All-cause death | 0.118 | 0.004 |
| Stroke | 0.175 | 0.045 |
| Any serious adverse event | 0.806 | 0.465 |
| Infection | 0.734 | 0.678 |
| Any cancer | 0.221 | 0.245 |
